# Asn-Gly-Arg-modified polydopamine-coated nanoparticles for dual-targeting therapy of brain glioma in rats

**DOI:** 10.18632/oncotarget.12047

**Published:** 2016-09-15

**Authors:** Jiangang Hu, Xiang Zhang, Zuhuang Wen, Ying Tan, Ning Huang, Si Cheng, Huzhi Zheng, Yuan Cheng

**Affiliations:** ^1^ Department of Neurosurgery, the Second Affiliated Hospital of Chongqing Medical University, Chongqing, 400010, China; ^2^ Chongqing Key Laboratory of Ultrasound Molecular Imaging, Institute of Ultrasound Imaging of Chongqing Medical University, Chongqing, 400016, China; ^3^ College of Chemistry and Chemical Engineering, Southwest University, Chongqing, 400715, China; ^4^ Department of Orthopaedics, the First Affiliated Hospital of Chongqing Medical University, Chongqing, 400016, China

**Keywords:** Asn-Gly-Arg, polydopamine, dual-targeting, gliomas

## Abstract

The blood-brain barrier (BBB) is the major clinical obstacle in the chemotherapeutic management of brain glioma. Here we synthesized a pH-sensitive dual-targeting doxorubicin (DOX) carrier to compromise tumor endothelial cells, enhance BBB transportation, and improve drug accumulation in glioma cells. The drug delivery system was constructed with polydopamine (PDA)-coated mesoporous silica nanoparticles (NPs, MSNs) and the PDA coating was functionalized with Asn-Gly-Arg (NGR), a ligand with specific affinity for cluster of differentiation 13 (CD13). MSN-DOX-PDA-NGR showed a higher intracellular accumulation in primary brain capillary endothelial cells (BCECs) and C6 cells and greater BBB permeability than the non-targeting NPs (MSN-DOX-PDA) did *in vitro*. *Ex vivo* and *in vivo* tests showed that MSN-DOX-PDA-NGR had a higher accumulation in intracranial tumorous tissue than the undecorated NPs did. Furthermore, the antiangiogenesis and antitumor efficacy of MSN-DOX-PDA-NGR were stronger than that of MSN-DOX-PDA. Therefore, these results indicate that the dual-targeting vehicles are potentially useful in brain glioma therapy.

## INTRODUCTION

The delivery of chemotherapeutic agents for the treatment of central nervous system diseases such as brain glioma is still a major challenge because most chemotherapy drugs cannot penetrate the blood brain barrier (BBB) to reach the brain tumor cells [1, 2]. Numerous attempts have been made to overcome this barrier, but trans-BBB drug delivery is still a challenge. Recently, an efficient receptor-mediated, stimuli-responsive controlled drug release system for penetrating the BBB and targeting brain tumor cells has attracted considerable attention [3-6].

As a marker for angiogenic vasculature, the aminopeptidase N (cluster of differentiation 13, CD13) is overexpressed in cancerous tissues including neovascular endothelial cells and various tumor cells and has been widely studied in tumor angiogenesis and invasion [7]. A peptide containing the Asn-Gly-Arg (NGR) motif, which binds CD13 has also been associated with chemotherapeutic drug delivery [8-10]. However, targeting and compromising glioma cells remain challenging even with these delivery systems. Therefore, we designed an inorganic-organic hybrid nanoparticle (NP)-based dual-targeting carrier (tumor vascular- and tumor-targeting), incorporating the advantages of mesoporous silica NPs (MSN) and polydopamine (PDA).

MSN are ideal nanocarriers for chemotherapy drugs because of their low density, large surface area, high pore volume, high drug-loading capacity, and excellent biocompatibility [11-14]. A key step in constructing the controlled drug release carrier was the generation of “gatekeepers” on the MSN surface to seal the drug molecules inside the mesoporous core and control the drug release from MSN in response to the pH-stimulus [15]. PDA, a mussel adhesive protein mimetic, has the potential to act as the pH-sensitive gatekeeper and conjugate the NGR to enhance targeting, owing to the versatility in modifying [16], easiness in surface functionalization with NGR motif via Michael addition reactions [17, 18], negligible cytotoxicity in drug delivery [19, 20].

Therefore, for the first time, we synthesized a controlled release and dual-targeting system using PDA-coated MSN as the doxorubicin (DOX) carrier and conjugating NGR as the targeting ligand (MSN-DOX-PDA-NGR) to dual-target the endothelial and glioma cells. Primary rat brain capillary endothelial cells (BCECs) and C6 cells were chosen as the model of tumor endothelial and tumor cells, respectively. The drug delivery system was characterized using transmission electron microscopy (TEM) and dynamic light scattering (DLS). The drug release behavior, cell cytotoxicity, uptake assays, and dual-targeting effects were evaluated *in vitro* while the antiangiogenesis and antitumor effects were investigated *in vivo*.

## RESULTS

### Characterization of dual-targeted NPs

**Figure 1:**
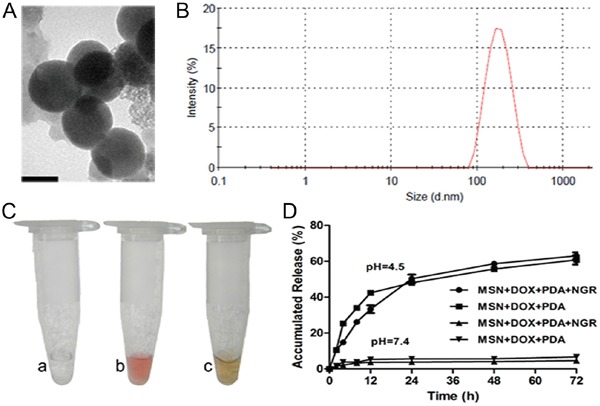
Characterization of dual-targeted NPs **A.** Transmission electron microscopy (TEM) images of MSN-DOX-PDA-NGR NPs. **B.** Particle size distribution of MSN-DOX-PDA-NGR NPs. **C.** Color change of NPs solution. **D.** *In vitro* release profiles of DOX from different drug carriers in different PBS samples at pH (4.5) and pH (7.4). Scale bar, 100 nm.

NPs with the size of 100-200 nm are believed to have the most potential for persistent circulation and exhibit the best enhanced permeation and retention (EPR) effect [21]. Therefore, we selected MSN with a mean particle diameter of 150 nm to prepare the MSN-DOX-PDA-NGR. DOX was first loaded into the silica mesopores and then coated with PDA via oxidative self-polymerization of dopamine. The surface of the MSN-DOX-PDA NPs was then modified with NGR via a Michael addition. The TEM images showed that the NPs were spherical and had a smooth surface with a size of approximately 160 nm. The dehydration and shrinkage of the NPs during the preparation for TEM observation may lead to the smaller diameter compared with the results from DLS (Table 1 and Figure 1A). The NGR modification slightly increased the particle size and zeta potential (Table 1 and Figure 1B). It has been indicated that the negatively-charged surface of the drug carrier system has higher blood compatibility and better targeting effect *in vivo* than the positively-charged carrier surface does [22, 23]. The loading of DOX and the modification of the PDA coating were also reflected in the color change of the solution. The white-colored MSN solution turned brick red after DOX loading and subsequently turned dark brown after the modification of the PDA coating (Figure 1C). The loading content (LC), encapsulation efficiency (EE), and NGR conjugation efficiency (CE) values were 19.02%, 40.84%, and 41.6%, respectively, under the present experimental conditions.

**Table 1:** Size and zeta potential of nanoparticles (NPs, n = 3)

	Size (nm)		Zeta potential (mv)
	TEM	DLS	
MSN-DOX-PDA	152.3 ± 0.97	156.1 ± 1.25	−24 ± 0.96
MSN-DOX-PDA-NGR	160.1 ± 1.49	168.0 ± 1.32	−22 ± 0.97

Because most tumorous tissues have low extracellular pH values, especially inside the endosomal (pH, 5.5–6.0) and lysosomal (pH, 4.5–5.0) compartments [24, 25], we conducted the DOX release profiles at pH 4.5 and 7.4. As shown in Figure 1D, only a very small amount of DOX was released in the neutral PBS (pH 7.4). However, the release rate dramatically improved and the cumulative release rate of DOX was approximately 50% within 24 h at pH 4.5. The pH-sensitivity of the carriers suggested that the NPs were almost stable in plasma conditions but allowed sustained drug release in tumorous conditions, which facilitated drug accumulation in the tumorous vasculature and intercellular distribution in tumor cells.

### Receptor expression level in targeted cells

**Figure 2:**
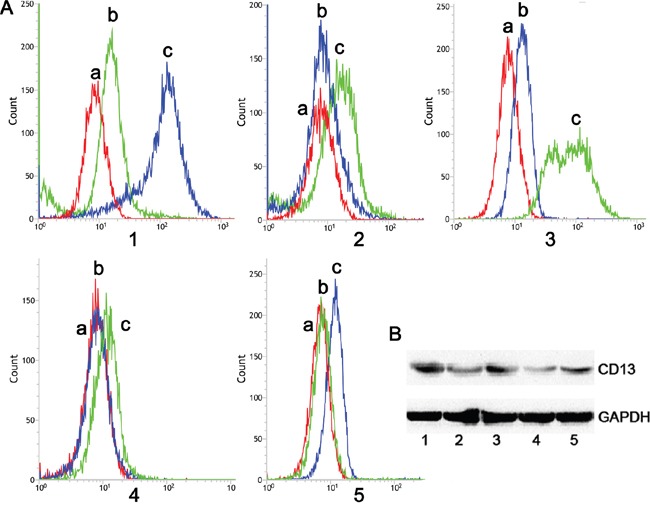
Flow cytometry and western blot analysis of NP-treated cells **A.** Flow cytometry and **B.** western blotting analysis of CD13 protein expressed in (1) BCEC-C6, (2) BCEC, (3) C6, (4) astrocytes, and (5) BCEC-AC cells; (a) negative control, (b) mouse IgG2a K Isotype control, and (c) anti-rat CD13 antibody (FITC).

CD13 is expressed exclusively on the angiogenic endothelial and not normal vasculature [26]. In addition, the high level of vascular endothelial growth factor (VEGF) released by C6 cells strikingly could upregulate the CD13 level in primary cells during angiogenesis [27, 28]. Therefore, we established a C6 and primary brain capillary endothelial cells (BCEC) cocultured model to investigate whether the CD13 level in BCEC cells was upregulated. The flow cytometry and western blotting showed a dramatic elevation of the CD13 level in BCECs cocultured with C6 cells (BCEC-C6). In addition, the level was relatively higher in C6 cells than it was in the primary astrocytes (AC), BCEC, and BCECs cocultured with AC cells (BCEC-AC) (Figure 2A and 2B). The results indicate that C6 cells successfully induced the BCECs and the tumorous vasculature endothelial cell model was established.

### Cellular uptake of dual-targeted NPs

**Figure 3:**
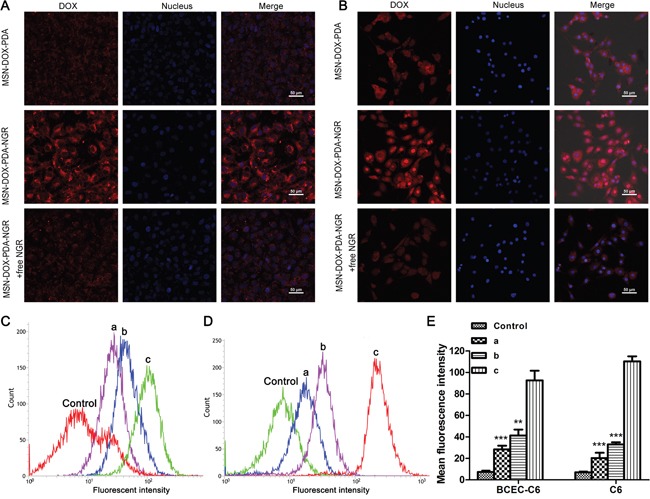
Qualitative and quantitative analysis of drug-loaded NPs in BCEC-C6 and C6 cells Confocal images of **A.** BCEC-C6 and **B.** C6 cells after treatment with different DOX formulations for 4 h. Flow cytometric analysis of **C.** BCEC-C6 and **D.** C6 cells after treatment with different DOX formulations for 2 h. **E.** Quantitative analysis of mean fluorescence intensity in different DOX formulation group. Cell nuclei were stained using DAPI (blue) and DOX (red). (a) MSN-DOX-PDA, (b) MSN-DOX-PDA-NGR + free NGR, and (c) MSN-DOX-PDA-NGR. ^**^p < 0.01 and ^***^p < 0.001 compared with c (n = 3).

To determine the specific recognition and uptake capacity of MSN-DOX-PDA-NGR towards target cells, we performed laser scanning confocal microscopy and flow cytometry on C6 and BCEC-C6 cells after treatment with different DOX formulations. Both C6 and BCEC-C6 cells in the MSN-DOX-PDA-NGR group showed an obviously higher fluorescence intensity than those in MSN-DOX-PDA group did (Figure 3A and 3B). The identical results was also found by flow cytometry, the fluorescence intensity of the MSN-DOX-PDA-NGR group increased significantly compared with that of the undecorated NPs group (~3.27- and 5.50-fold for BCEC-C6 and C6, respectively). However, the adding of free NGR significantly inhibited the uptake of MSN-DOX-PDA-NGR (Figure 3C-3E). The NGR-mediated specific binding between MSN-DOX-PDA-NGR and glioma cells was also found in U251 cells but not in GL261 (Supplementary Figure S1). Furthermore, flow cytometry confirmed that, relatively to GL261, C6 and U251 have a more powerful binding affinity to anti-CD13 antibody (FITC) in our preliminary experiments (data not shown). Taken together, MSN-DOX-PDA-NGR specifically recognize and abundantly bind to BCEC-C6 and C6 cells through the high level of CD13.

### *In vitro* cytotoxicity of dual-targeted NPs

**Figure 4:**
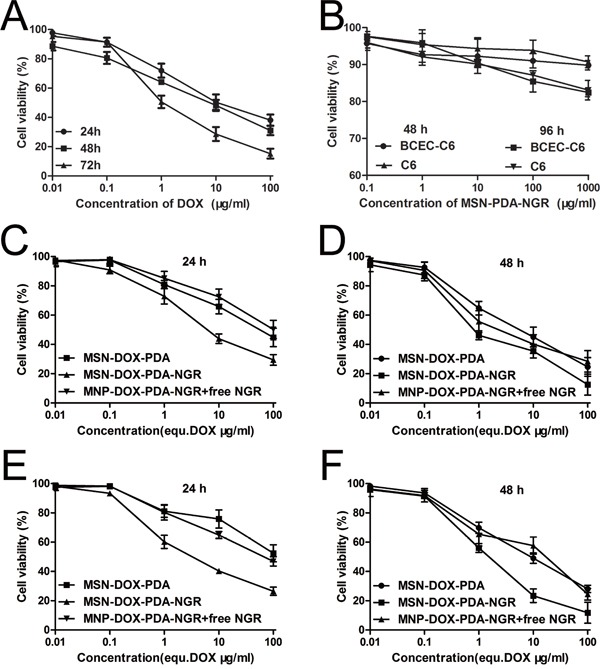
Cell viability following treatment with various NPs **A.** Viability of C6 cells after treatment with free DOX. **B.** Viability of BCEC-C6 and C6 cells after treatment with blank NPs. Viability of BCEC-C6 cells after treatment with different DOX formulations for **C.** 24 and **D.** 48 h. Viability of C6 cells after treatment with different DOX formulations for **E.** 24 and **F.** 48 h.

To evaluate the cytotoxicity of various DOX formulations against glioma cells with varying CD13 levels, the CCK-8 assay was carried out on C6, BCEC-C6, GL261 and U251 cells. For C6 cells, the cytotoxicity of the different DOX formulations was concentration- and incubation time-dependent. The IC_50_ values of DOX, MSN-DOX-PDA, MSN-DOX-PDA-NGR, and MSN-DOX-PDA-NGR + free NGR were 16.90, 138.1, 5.09, and 59.71 μg/mL after a 24-h incubation and 7.608, 9.85, 1.74, and 10.61 μg/mL after 48-h, respectively. NGR modification significantly decreased the IC_50_ values of the undecorated NPs (~27.13- 5.66-fold at 24 and 48 h, respectively). However, the addition of free NGR obviously increased the IC_50_ compared with that of MSN-DOX-PDA-NGR (~11.7- and 6.1-fold at 24 and 48 h, respectively) (Figure 4A, 4E, and 4F). This reversal was possibly attributable to the competition between the modified NGR on the NPs and the added free NGR. A similar reversal phenomenon was also displayed in the BCEC-C6 and U251 cells (Figure 4 and Supplementary Figure S2). However, unlike the perfect cytotoxicity observed in C6 cells, MSN-DOX-PDA-NGR did not exhibit an improved antitumor effect on GL261 cells compared with MSN-DOX-PDA and MSN-DOX-PDA-NGR + free NGR. This MSN-DOX-PDA-NGR resistance was evidenced by their no difference of IC_50_ values (*p* > 0.05) (Supplementary Figure S2C and S2D). The blank NPs were biocompatible since the cell viability was > 80% at a concentration of 1000 μg/mL of MSN-PDA-NGR and a 96-h treatment period (Figure 4B). In summary, owing to high CD13 level and subsequently MSN-DOX-PDA-NGR sensitization, C6 was selected as tumor cell model for the further studies.

### Cellular uptake mechanism of dual-targeted NPs

**Figure 5:**
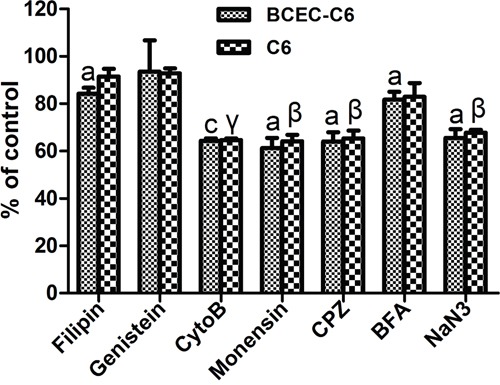
Effect of inhibitors on cellular uptake of MSN-DOX-PDA-NGR by BCEC-C6 and C6 cells ^a^*p* < 0.05 and ^c^*p* < 0.001 compared with control BCEC-C6 cells; ^β^*p* < 0.01 and ^γ^*p* < 0.001 compared with control C6 cells (n = 3).

To elucidate the endocytosis pathways of the MSN-DOX-PDA-NGR that were activated after their recognition by the CD13 expressed on glioma cells and endothelial cells, the effects of different endocytosis inhibitors were evaluated in C6 and BCEC-C6 cells. As shown in Figure 5, the uptake of MSN-DOX-PDA-NGR by C6 cells was significantly inhibited by NaN3 (energy depletion agent), Cyto-B (macropinocytosis inhibitor), CPZ (clathrin-mediated endocytosis inhibitor), and monensin (lysosome inhibitor), but not by filipin, genistein (caveolae-mediated endocytosis inhibitor) or BFA (Golgi apparatus and intracellular trafficking inhibitor). Furthermore, only genistein did not inhibit the uptake of MSN-DOX-PDA-NGR by the BCEC-C6 cells. These results indicate that the MSN-DOX-PDA-NGR uptake was energy-dependent and stimulated by clathrin-mediated endocytosis and the macropinocytosis pathway.

### Subcellular localization of dual-targeted NPs

**Figure 6:**
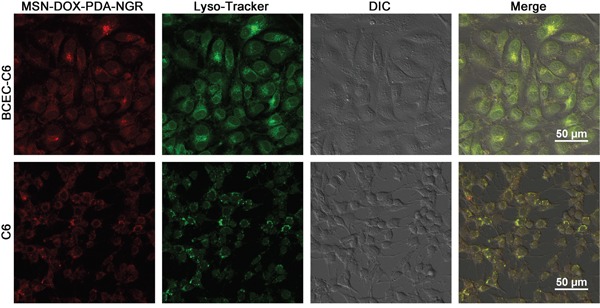
Confocal microscopy images of intracellular localization of MSN-DOX-PDA-NGR in A. BCEC-C6 and B. C6 cells Lysosomes and nanoparticles (NPs) were distinguished using Lysotracker (green) and DOX (red), respectively.

The results that the monensin (lysosome inhibitor) had an obvious inhibitory effect on the uptake of the NPs suggest that the uptake and release of NPs partly depended on the acidic environment in the lysosomes. To demonstrate this hypothesis directly, we examined the subcellular localization of the MSN-DOX-PDA-NGR in the C6 and BCEC-C6 cells using confocal laser scanning microscopy. The lysosomes showed green fluorescence after the cells were stained with organelle-selective dyes while the NPs showed red fluorescence. The colocalization of the NPs with the organelle-selective dyes was indicated by the yellow color in the superimposed image. As shown in Figure 6, the MSN-DOX-PDA-NGR was localized in the lysosomes of the C6 and BCEC-C6 cells, suggesting that following its uptake by the cells, the NPs was subsequently delivered to lysosomes.

### *In vitro* transendothelial permeability and dual-targeting of NPs in BBB model

**Figure 7:**
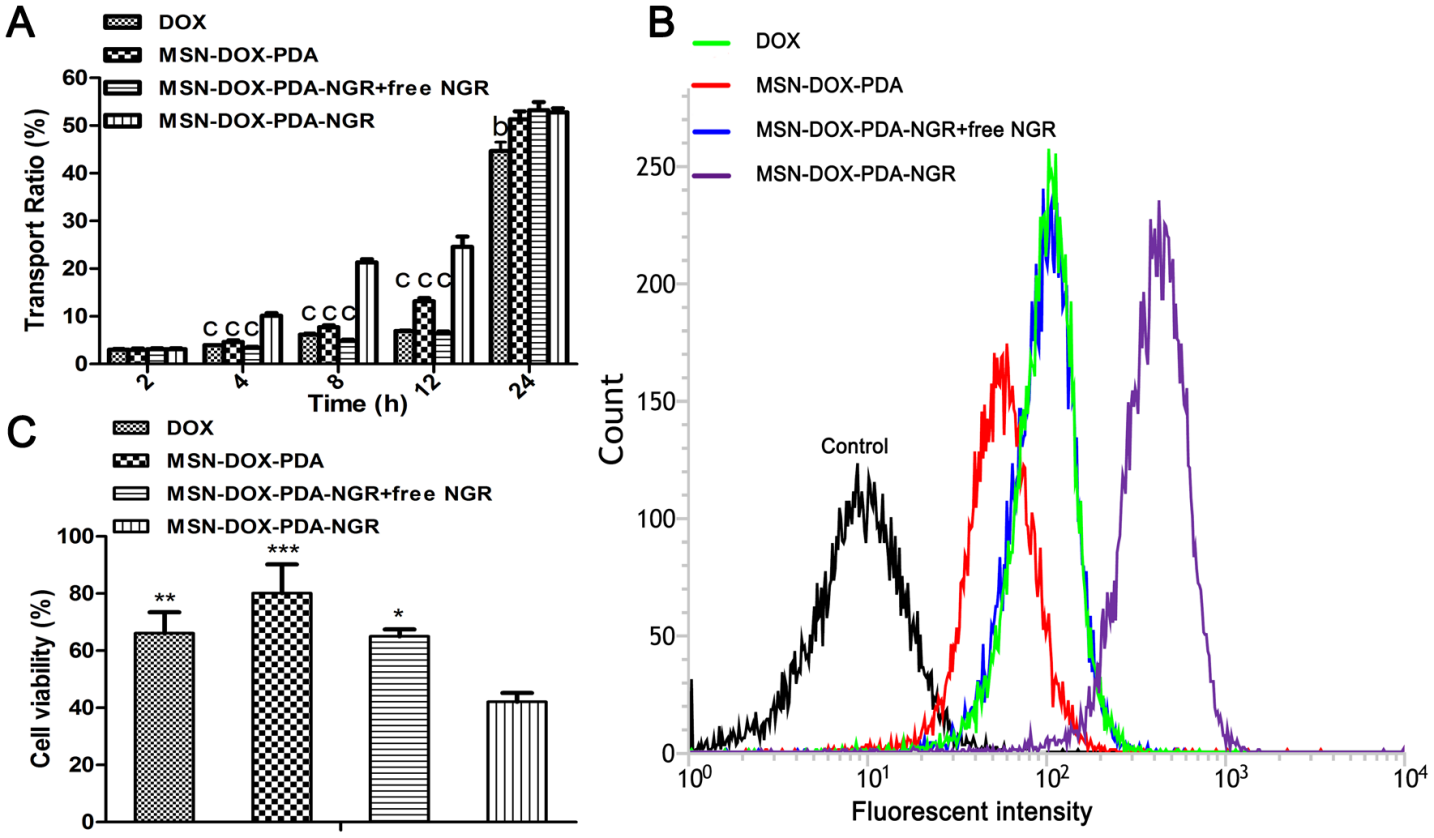
*In vitro* transport ratios (%) of various NPs **A.** Transport ratio across BCEC-C6 monolayer of different DOX formulations during 24 hours. **B.** Flow cytometric analysis and **C.** viability of C6 cells (below) after treatment with different DOX formulations; ^b^*p* < 0.01, ^c^*p* < 0.001, ^*^p < 0.05, ^**^p < 0.01, and ^***^p < 0.001 compared with MSN-DOX-PDA-NGR (n = 3).

The positive results of the experiments on the BCEC-C6 and C6 cells might not persuasively represent the dual-targeting capacity of MSN-DOX-PDA-NGR because of the extreme differences between the *in vitro* and *in vivo* conditions. Therefore, the *in vitro* BBB model was constructed to assess the transport efficiency of the different DOX formulations. Figure 7A shows that the *in vitro* transport ratios across the BBB increased with incubation time. After the 12-h incubation, the transport ratio was 13.16 and 24.59% for the MSN-DOX-PDA and MSN-DOX-PDA-NGR, respectively. In addition, the transport ratio for the MSN-DOX-PDA-NGR group was consistently the highest among these groups at different times, except 24 h. These results suggest that the transportation of the NPs across *in vitro* BBB model was significantly enhanced by the NGR modification (*p* < 0.001). However, when the excess NGR was pre-incubated to saturate the CD13 binding site of the *in vitro* BBB, the transport ratio of the MSN-DOX-PDA-NGR was significantly reduced. Therefore, it can be concluded that NGR might mediate the transport of MSN-DOX-PDA-NGR across the BBB via facilitative receptor-mediated transcytosis. The integrity of the BCEC monolayer was monitored by determining the trans-endothelial electrical resistance (TEER) during the entire experiment (data not shown), and no significant reduction was observed except the 24 h time point. This indicated that the drug transport did not compromise the BBB barrier properties, and the NPs transport across the BBB was via the transcellular but not the paracellular pathway within 12 hours. Surprisingly, at 24 h, the transport ratios of all the groups reached approximately 50%. These results may have been due to the breakdown of the BCEC monolayer as the TEER values were significantly reduced in all groups at the same time.

In addition, an *in vitro* coculture model was established to evaluate whether the drug-loaded NPs transported across the BBB could be taken up by the C6 cells below and induce cell death. Figure 7B and 7C show that the MSN-DOX-PDA-NGR group still exhibited the highest accumulation of DOX and cytotoxicity against the C6 cells. These results demonstrate that MSN-DOX-PDA-NGR exhibited a “dual-targeting effect,” which first targeted and transported across the BCEC monolayer and then targeted the C6 cells below.

### *In vivo* imaging of dual-targeted NPs

**Figure 8:**
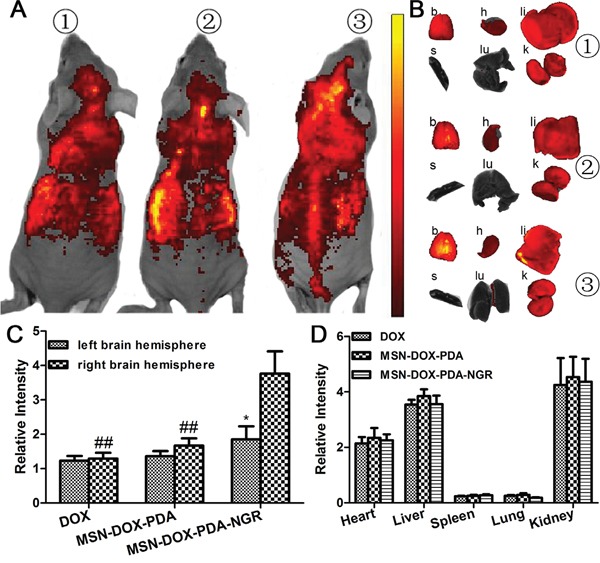
*In vivo* and *Ex vivo* fluorescent imaging of various NPs **A.** *In vivo* fluorescent imaging of different DOX formulations 6 h post i.v. injection. **B.** *Ex vivo* fluorescent imaging of dissected organs. Semi-quantitative fluorescence intensity of **C.** left and right brain hemisphere and **D.** other major organs. Mouse 1, injection of free DOX; mouse 2, injection of MSN-DOX-PDA; mouse 3, injection of MSN-DOX-PDA-NGR. Key: b, brain; h, heart; li, liver; s, spleen; lu, lung; k, kidney; ^*^p < 0.05 compared with right brain hemisphere and ^##^*p* < 0.01 compared with right brain hemisphere in MSN-DOX-PDA-NGR group (n = 3).

To evaluate the *in vivo* BBB penetration and NGR-targeting efficacy of different DOX formulations, non-invasive fluorescence imaging was conducted to track the biodistribution of NPs in C6 cell orthotopic glioma nude mice. A significantly higher fluorescence intensity at the brain tumor site was observed in the MSN-DOX-PDA-NGR group than in the MSN-DOX-PDA and free DOX groups (Figure 8A), indicating that MSN-DOX-PDA-NGR accumulated more in the glioma-related region. Moreover, the ratios of the right/left brain hemisphere fluorescence intensity were 1.05, 1.23, and 2.03 for DOX, MSN-DOX-PDA, and MSN-DOX-PDA-NGR, respectively (Figure 8B and 8C). In addition, the fluorescence intensity of the right brain in the MSN-DOX-PDA-NGR group was significantly higher than it was in the MSN-DOX-PDA and DOX groups. This observation indicates that MSN-DOX-PDA-NGR had an excellent dual-targeting delivery capability for glioma tissues. Furthermore, the distribution of the different DOX formulations in the other investigated organs was comparable (Figure 8B and 8D).

### *In vivo* glioma distribution of dual-targeted NPs

**Figure 9:**
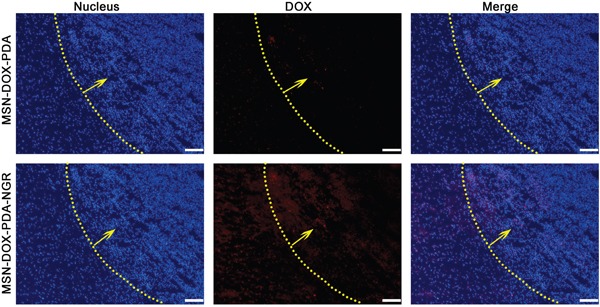
*In vivo* distribution of different DOX formulations in brains of glioma-bearing rats Nuclei were stained with DAPI (blue) while DOX distribution is in red. Yellow line and arrow, border and direction of glioma, respectively; scale bar, 50 μm.

We sought to further determine whether MSN-DOX-PDA-NGR specifically targeted the tumor cells *in vivo*. Therefore, we studied the glioma-targeting capacity of the MSN-DOX-PDA-NGR qualitatively using fluorescence microscopy in tumor bearing rats. As shown in Figure 9, only a few MSN-DOX-PDA NPs were distributed in the glioma region owing to the EPR effect. However, an obviously higher distribution of the NGR-modified NPs was observed at the glioma site and in the cancerous brain tissues surrounding the glioma than in the normal regions. These findings were consistent with the *ex vivo* fluorescence images of the NPs distribution in glioma-bearing nude mice.

### *In vivo* drug release and liver deposition

**Figure 10:**
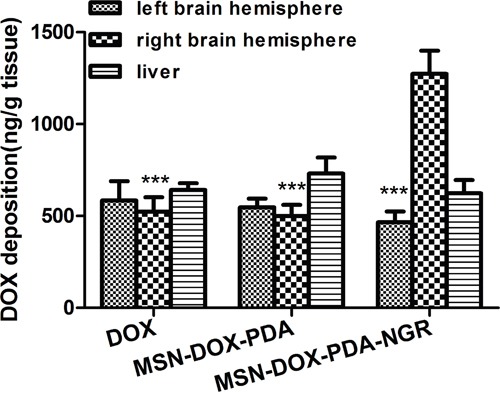
DOX deposition in tumor-bearing brain tissue, contralateral brain tissue, and liver tissue after treatment with different DOX formulations ^***^p < 0.001 compared with the right brain hemisphere in MSN-DOX-PDA-NGR group (n = 3).

To evaluate the drug delivery capacity of the NPs in tumor bearing rats quantitatively, the tumor-bearing and contralateral brain tissues, as well as liver tissues, were harvested after treatment with the different DOX formulations, and the amount of DOX was measured using HPLC analysis (Figure 10). The DOX deposition in the right brain hemisphere of the MSN-DOX-PDA-NGR group was significantly greater than that of the MSN-DOX-PDA and DOX groups (2.55 and 2.43-fold, respectively). The amount of DOX in the right and left brain hemispheres showed no obvious difference between the DOX and MSN-DOX-PDA groups. However, the DOX deposition was significantly higher in the tumor-bearing brain tissue than it was in the contralateral normal brain tissue in the MSN-DOX-PDA-NGR group (*p* < 0.001). These results are in accordance with those of the *ex vivo* imaging and *in vivo* glioma distribution of the dual-targeted NPs. Moreover, the DOX deposition in liver tissues showed no significant difference among the three groups.

### *In vivo* antitumor and antiangiogenesis effects of dual-targeted NPs

**Figure 11:**
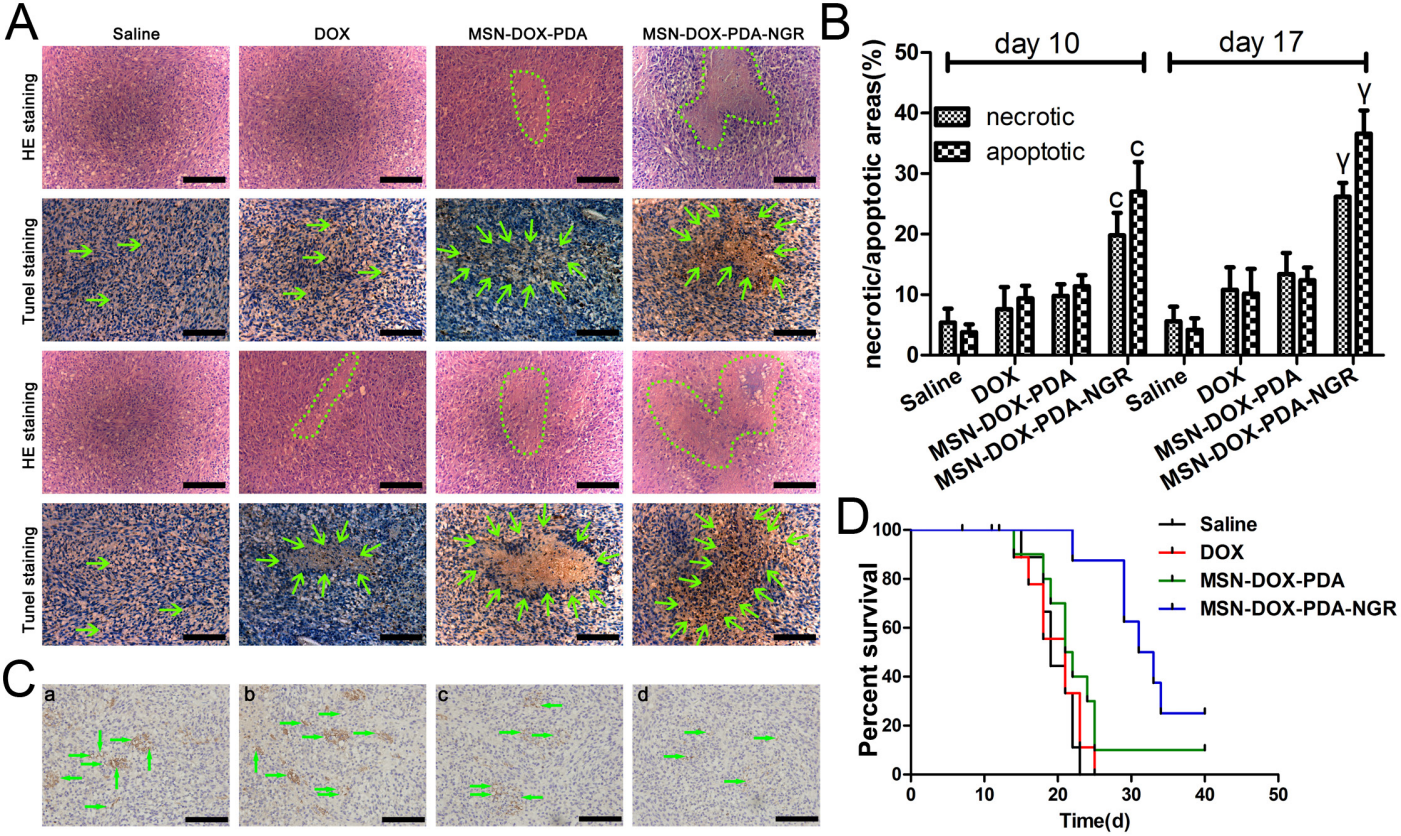
*In vivo* antitumor and antiangiogenesis effects of dual-targeted NPs **A.** H&E and TUNEL staining after 10 and 17 days (upper and lower two rows, respectively) post transplantation. **B.** The ratios of the necrotic/apoptotic areas within the tumor. **C.** CD31-positive microvessels in glioma-bearing rat brains. **D.** Survival curve (Kaplan-Meier plot) of glioma-bearing rats treated with different DOX formulations. Green circle and arrows in A indicate necrotic regions and cell apoptosis body in tumor interior, respectively; a, control group; b, free DOX group; c, MSN-DOX-PDA group; d, MSN-DOX-PDA-NGR group. Green arrow in C, microvessel; scale bar, 50 μm. ^c^*p* < 0.001 compared with other groups at day 10; ^γ^*p* < 0.001 compared with other groups at day 17 (n = 5).

We assumed that the dual-targeted NPs have a more powerful antitumor activity since the MSN-DOX-PDA-NGR increased the DOX accumulation at the tumor site. To evaluate the antitumor effect of the MSN-DOX-PDA-NGR *in vivo*, we examined the apoptotic and necrotic regions in the brains of tumor bearing rats using H&E staining and TUNEL assay after treatment with different DOX formulations. As shown in Figure 11A and 11B, obvious apoptotic and necrotic tumor cells were observed in MSN-DOX-PDA-NGR group on day 10 compared with those in other groups. In addition, a more severe cell apoptotic and necrotic was detected on day 17, suggesting that NGR-decoration NPs could not only target but also put down the tumor cells *in vivo*.

To determine the antiangiogenic activity simultaneously, we assessed the microvessel density using immunohistochemistry. As shown in Figure 11C, the microvessels in the glioma-bearing rat brains were CD31-positive. There were obviously fewer microvessels in the MSN-DOX-PDA-NGR group than there were in other groups. The results were in accordance with the H&E staining and TUNEL assay, clearly indicating that MSN-DOX-PDA-NGR possesses dual-targeting capacity *in vivo*.

To further evaluate the therapeutic activity *in vivo*, the survival time of the tumor-bearing rats after treatment was investigated using the Kaplane-Meier survival curve. As shown in Figure 11D, the median survival times for the saline, DOX, MSN-DOX-PDA, and MSN-DOX-PDA-NGR groups were 19, 21, 21.5, and 32 days, respectively. Compared with the saline group, the DOX and MSN-DOX-PDA did not show an improvement in the survival time. However, treatment with MSN-DOX-PDA-NGR significantly prolonged the median survival time compared with that for other groups.

MSN-DOX-PDA-NGR showed significant antitumor and antiangiogenesis effects and obviously prolonged the survival time compared with the other groups. These observations suggest that the MSN-DOX-PDA-NGR with dual-targeting characteristics possessed the most effective therapeutic activity of all the formulations investigated.

### *In vivo* biological safety study

**Figure 12:**
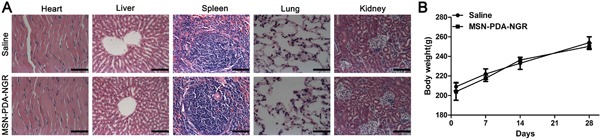
*In vivo* biological safety study **A.** Hematoxylin and eosin (H&E) staining of major organs harvested from rats treated with MSN-PDA-NGR for 4 weeks. **B.** Body mass of rats after treatment with MSN-PDA-NGR.

The potential *in vivo* toxicity of NPs for drug delivery is always of great concern. For safety purpose, we evaluated the *in vivo* toxicity of the drug carrier in healthy adult male rats treated with MSN-PDA-NGR by tail vein injections at one 100 mg/kg dose administered every 3 days for 4 weeks. The increase in body mass of the MSN-PDA-NGR and saline groups showed a comparable tendency over the 4 weeks. Furthermore, we conducted the histological analyses, which revealed no obvious pathological lesions or impairment in the major tissues from rats that received MSN-PDA-NGR for 4 weeks (Figure 12).

## DISCUSSION

Dual-targeting drug delivery systems that target some overexpressed receptors on both tumor neovasculature and cells have been developed. These systems offer a great strategy for overcoming the limitation of current antiangiogenesis therapy by combining vasculature damage and traditional anticancer therapy [29]. The main objective of this study was to verify the hypothesis that NGR-modified drug delivery systems could not only kill brain tumor angiogenic blood vessels, which indirectly starve the tumor cells supported by these vessels, but also penetrate the brain tumor interstitial space, thereby inducing direct tumor cell death. Briefly, NGR-conjugated NPs (MSN-DOX-PDA-NGR) could serve as a promising dual-targeting drug carrier for brain glioma and neovasculature chemotherapy.

Recent studies have shown that MSN employed as a targeting carrier possesses low cytotoxicity and good biocompatibility when applied to mammalian cells [30, 31]. Dopamine is a well-known neurotransmitter. However, it has been demonstrated that its monomer does not exist in the PDA coating, which has been shown to be non-toxic in various cellular and *in vivo* studies [32, 33]. In our experiments, we also found that the *in vitro* cytotoxicity and *in vivo* toxicity of MSN-PDA-NGR were negligible. In summary, the evidence provided in these experiments suggests that our dual-targeting system is suitable for drug delivery.

For the *in vitro* experiments, the cellular uptake of the NPs and the subsequent cytotoxicity were enhanced by NGR modification, as demonstrated by the confocal images, flow cytometric analysis, and CCK-8 assay. To mimic *in vivo* conditions, the *in vitro* BBB model was constructed to assess the transport efficiency of the different DOX formulations. MSN-DOX-PDA-NGR yielded the most efficient BBB transport ratio and subsequent cytotoxicity in the C6 cells before the integrated BBB destruction. The superior BBB permeability of the MSN-DOX-PDA-NGR may have contributed to the combination of the receptor-mediated cellular uptake and the caveolae- and clathrin-mediated endocytosis. After the BBB was perturbed by the chemotherapy drugs in the donor chamber, a vast amount of the DOX formulations passed through the disrupted barrier and accumulated in the “tumorous tissue fluid” in basal chamber. In agreement with the promising results of the *in vitro* model, the *in vivo* trans-endothelial superiority was demonstrated qualitatively by the *ex vivo* biodistribution and *in vivo* glioma distribution of the MSN-DOX-PDA-NGR, and quantitatively by the *in vivo* DOX deposition in the glioma-bearing rat brain.

Angiogenesis and vascular proliferation are regarded as critical factors for tumor growth, progression, and invasion and are established hallmarks of the malignancy degree of gliomas [34]. Numerous tumor cells depend on endothelial cells for their survival and invasion. Therefore, the method that compromised the tumor endothelial cells used in our experiment may amplify the therapeutic effect. The positive results of the *in vivo* immunohistochemical analysis of CD31 and histopathological changes in the glioma tissue verified the antiangiogenesis and antitumor effects of MSN-DOX-PDA-NGR, respectively. The prolonged median survival time of the MSN-DOX-PDA-NGR-treated tumor-bearing rats further confirmed this amplified therapeutic effect. Overall, it is clear that the dual-targeting drug carrier did not only deliver the chemotherapy drugs into the tumor endothelial and glioma cells more efficiently, but it also induced more obvious cell apoptosis and necrosis. This observation indicates that the MSN-PDA-NGR NPs are a promising carrier for dual-targeted drug delivery. Since a higher CD13 level in the glioma (WHO grade III-IV) was detected than that in the gliom (I-II) using immunohistochemistry in our preliminary study (data not shown), we assumed that the MSN-DOX-PDA-NGR exhibited more powerful antitumor effects against the high-grade glioma than against low-grade. Therefore, we plan to establish different grade human primary glioma animal model to evaluate the therapeutic effects of the NPs in the further study.

## MATERIALS AND METHODS

### Materials and animals

Mesoporous silica with a mean particle diameter of 150 nm was purchased from Nanjing XFNANO Materials Tech Co., Ltd., (Nanjing, China). The dopamine hydrochloride was purchased from Alfa Aesar (Ward Hill, MA, USA) while the NGR was synthesized by the Chinese Peptide Company (China). The DOX and all other chemicals were purchased from Sigma-Aldrich (St. Louis, MO, USA) unless otherwise specified. The plastic cell culture dishes and plates were purchased from Corning Inc., (Corning, NY, USA). The anti-CD31 and anti-CD13 antibodies were purchased from Abcam (MA, USA) while the anti-CD13 (fluorescein isothiocyanate, FITC) was purchased from Biorbyt (Cambridgeshire, UK). The primary BCEC cultures and astrocytes cells (AC) were prepared from 4-week- and 3-day-old Sprague-Dawley male rats, respectively, provided by the Experimental Animal Center of Chongqing Medical University. The C6, GL261, U251 cells were obtained from the Chinese Academy of Sciences Cell Bank (Shanghai, China). All animal experiments were performed according to the protocol evaluated and approved by the Ethics Committee of Chongqing Medical University.

### Preparation and characterization of dual-targeted NPs

The MSN (12 mg) was added to 6 mL DOX solution (1 mg/mL) and the mixture was stirred in the dark for 24 h at 25°C. Then, the DOX-loaded MSNs (MSN-DOX) were separated by centrifugation (12000 rpm, 10 min), and washed with water to remove the residual DOX from the exterior surface of MSN. The filtrate and the wash solution were collected and combined as the remaining DOX solution. The loading amount of DOX was calculated as the difference between the initial and remaining DOX solution by using the fluorescence method using a calibration curve. Finally, the MSN-DOX were dried at 50°C under high vacuum overnight. The PDA coating was applied to modify the surface of the MSN as reported previously [15].

For the surface functionalization, the PDA-coated MSN-DOX (MSN-DOX-PDA) were resuspended in Tris buffer (10 mM, pH 8.5) containing NGR. The final concentrations of the NPs and ligands were 2 and 0.5 mg/mL, respectively. After 3-h incubation at 25°C with rotation, the NPs were collected by centrifugation (12000 rpm, 10 min), washed with water twice. The concentration of Asn-Gly-Arg (NGR) in the supernatant was determined using a high-performance liquid chromatography (HPLC) system (LCMS-2010, Shimadzu, Japan) equipped with a C18 column (4.6 mm × 250 mm, pore size 5-μm, Waters). Mobile phase, acetonitrile and water in a ratio of 70:30 (v/v), pH 3.08; flow rate, 1.0 mL/min; sample injection volume, 10 μL; detection wavelength, 220 nm. The encapsulation efficiency (EE) and loading content (LC) of drug, and the NGR conjugation efficiency (CE) were determined as follows: EE = DOX _encapsulated_/DOX_total_ × 100, LC = DOX_encapsulated_/materials × 100, CE = NGR _conjugated_/NGR _total_ × 100.

The particle size and zeta potential of NPs were determined using dynamic light scattering (DLS) with a Zeta Sizer 3000 HS (Malvern, USA). The morphology of the nanomaterial samples was observed using a transmission electron microscope (H-7600, Hitachi, Japan) at an acceleration voltage of 100 kV.

The drug release experiment was performed by diluting NP-DOX with PBS at pH 7.5 and acetate buffer at pH 4.5 at a concentration equivalent to 140 μg/mL free DOX, and incubating at 37°C for 2, 4, 8, 12, 24, 48, and 72 h. After incubation and centrifugation at 20000 rpm to remove the NPs, the concentration of DOX was determined using HPLC analysis (mobile phase, acetonitrile:water, 70:30 (v/v), pH 3.08; flow rate, 1.0 mL/min; sample injection volume, 5 μL; detection wavelength, 233 nm).

### CD13 expression in targeted cells

The BCECs, C6, and AC cells were seeded at a density of 5 × 10^5^ cells/dish. To promote the differentiation of the BCECs, coculture models were established with C6 or AC cells using Millicell hanging cell culture inserts (1-μm pore size, 24 mm diameter, 4.5 cm^2^ surface area, Millipore, USA). The BCECs were seeded in the six-well culture plates while the C6 or AC cells were in the donor chamber, and the cells were at a density of 1 × 10^5^ cells/well. When the cell confluency had attained 80% after 48 h, the C6 cells, AC cells, BCECs, and BCEC-C6 cells, BCEC-AC cells were harvested, incubated with the anti-CD13 antibody (FITC), mouse IgG2a K Isotype Control according to the manufacture's instruction. Then, the cells were analyzed using a fluorescence-activated cell sorting (FACS) Calibur flow cytometer (Becton Dickinson, Mountain View, CA, USA). The western blot analysis was further performed to quantify the CD13 levels in the single and cocultured cells according to a previously described method [35].

### Cellular uptake of dual-targeted NPs

For the qualitative study, a laser scanning confocal microscope (Leica SP2, Heidelberg, Germany) was used to investigate the uptake of NPs into C6 cells. The cells were cultured overnight at a density of 4 × 10^4^ cells/confocal dish and then treated with DOX-MSN-PDA, MSN-DOX-PDA-NGR, or MSN-DOX-PDA-NGR + free NGR at a DOX concentration of 0.5 μg/mL for 4 h. Then, the cells were rinsed thrice with phosphate-buffered saline (PBS), fixed with 4% (v/v) paraformaldehyde for 15 min, stained with 4′, 6-diamidino-2-phenylindole (DAPI) for 5 min, and finally washed thrice with PBS before observation.

For the quantitative study, flow cytometry was used to measure the fluorescence intensity of DOX at excitation and emission wavelengths of 488 and 585 nm, respectively. In brief, the C6 cells were treated with the different DOX formulations used in the quantitative study for 2 h in the dark, rinsed with PBS, and then harvested for detection. The method used to assess the cellular uptake of the NPs in the BCEC-C6, GL261, and U251 cells was the same as described above.

### Cytotoxicity assay of dual-targeted NPs

The C6 cells were seeded into 96-well culture plates at a density of 5 × 10^3^ cells/well the night before treatment. Then, free DOX, blank MSN-PDA-NGR, MSN-DOX-PDA, MSN-DOX-PDA-NGR, or MSN-DOX-PDA-NGR + free NGR with different concentrations were added to the wells of the 96-well plates and incubated together. At designated time points, 10 μL of the CCK-8 solution was added to each well followed by incubation for 2 h. The absorbance values (A_450nm_) were detected using a microplate reader (BioRad, 680, USA). The cell viability was calculated as follows: survival (%) = (A_450nm_ of treated cells/A_450nm_ of control cells) × 100. The half-maximal inhibitory concentration (IC_50_) values were determined from the dose-effect curve and expressed as the concentration of DOX-equivalency. For the BCEC-C6 cells, the cytotoxicity of the different NPs was also determined in the BCEC-C6, GL261, and U251 cells using the same method described above.

### Cellular uptake mechanism of dual-targeted NPs

The cellular uptake mechanism of dual-targeted NPs was performed as previously described [36]. The C6 cells were seeded at a density of 5×10^5^ cells/well into six-well plates and incubated overnight. After a 30-min incubation with Dulbecco's modified Eagle's medium (DMEM), the cells were further incubated with the following inhibitors, sodium azide (NaN_3_, 0.1%), filipin (10 μg/mL), chlorpromazine hydrochloride (CPZ, 20 μg/L), cytochalasin B (Cyto-B, 40 μM), monensin (100 nM), and brefeldin A (BFA, 20 μg/mL) for another 30 min. Then, the inhibitors were removed from the wells, and MSN-DOX-PDA-NGR (0.5 μg/mL) and fresh samples of the inhibitors at the same concentrations used previously were added. After 1-h incubation, the cells were harvested by trypsinization and analyzed using flow cytometer. Cells that were incubated without any inhibitors served as the control. A similar method was also used to evaluate the uptake mechanism in the BCEC-C6 cells.

### Subcellular localization of dual-targeted NPs

The subcellular localization of dual-targeted NPs was performed as previously described [36]. The C6 cells were cultured overnight in the confocal dish and treated with MSN-DOX-PDA-NGR (0.5 μg/mL) for 30 min at 37°C followed by incubation with 50 nM LysoTracker Green (30 min) and 10 mM Hoechst 33342 (10 min) to visualize the lysosome and nuclei using a laser scanning confocal microscope. A similar method was also used to evaluate the subcellular localization in the BCEC-C6 cells.

### Transport across BBB and dual-targeting effects of NPs *in vitro*

The transport ratio (%) was measured to assess the permeability of the drug carriers through the BBB. The BCECs were seeded into the Millicell hanging cell culture insert at a density of 1 × 10^5^ cells/well and cocultured with the C6 cells for 7 days, supplemented with hydrocortisone (500 nM) and cyclic adenosine monophosphate (cAMP, 312.5 μM) to induce barrier tightness [37-39]. Before starting the experiment, the tightness of the BCEC monolayer was monitored by measuring the trans-endothelial electrical resistance (TEER) using a TEER instrument (Millicell-RES, Millipore, USA). Only BCEC monolayers with TEER above 200 Ω cm^2^ were selected for the subsequent experiment [40]. Then, the BCEC monolayer described above was inserted into another six-well culture plate containing fresh medium, and different DOX formulations (free DOX, DOX-MSN-PDA, MSN-DOX-PDA-NGR, and MSN-DOX-PDA-NGR + free NGR) were added to the donor chamber at a DOX concentration of 100 μg/mL, followed by incubation at 37°C. A 300-μl sample was withdrawn from the acceptor chamber at 2, 4, 8, 12, and 24 h and an equal volume of fresh DMEM was added immediately after each sampling. The concentration of DOX was determined using an HPLC analysis. The transport ratio was calculated using the following formula: ratio (%) = (W_n_/W) × 100; where, W_n_ is the DOX amount in the samples taken from basal chamber at the nth h (n = 2, 3, 8, 12, and 24), and W is the DOX amount in the control added to the apical chamber. To assess the dual-targeting effect of the carriers *in vitro*, the BCEC monolayer was inserted into the six-well culture plate where the C6 cells had been cultured overnight at a density of 1 × 10^5^ cells/well and they were incubated together for 24 h to establish the coculture model *in vitro* [41]. Then, different DOX formulations were added to the apical chamber at a final DOX concentration of 100 μg/mL. For the cellular uptake, the C6 cells in the lower chamber were harvested for flow cytometric analysis after 8 h co-incubation. For the cell survival evaluation, the C6 cells were further cultured alone for 48 h and then measured using a CCK-8 assay. At the end of the experiment, the TEER was measured again to check the integrity of the BCEC monolayers.

### *In vivo* imaging of dual-targeted NPs in glioma-bearing nude mice

The C6 cell orthotopic glioma nude mouse model was established by injecting C6 cells (1 × 10^6^ cells in 10 μL PBS, 0.01 M, pH 7.4) into the right brain of male nude mice (2 mm lateral to the bregma and 5.0 mm deep from the dura) at a rate of 1.0 μL/min using a stereotaxic apparatus. The growth of the tumor was monitored longitudinally using magnetic resonance imaging (MRI). On day 10 after the implantation, the different DOX formulations were injected via the tail vein at a dose of 100 mg/kg body weight. Then, 6 h post-injection, the *in vivo* distribution of the associated fluorescence was visualized using the IVIS animal imaging system (PerkinElmer, USA). Then, 24 h post-injection, the *ex vivo* images of the major organs were also captured after they were harvested and rinsed with PBS thrice.

### *In vivo* glioma distribution of dual-targeted NPs

The glioma-bearing rat model was established using the method described above. Then, 8 h after treatment with DOX-MSN-PDA and MSN-DOX-PDA-NGR, the rats were anesthetized by the intraperitoneal (ip) administration of 5% chloral hydrate, followed by heart perfusion with saline and 4% paraformaldehyde. Subsequently, the brains were removed, fixed in 4% paraformaldehyde for 24 h, and sequentially cryoprotected with 15 and 30% sucrose solutions until subsidence was observed with each exposure. Then, the brains were frozen in optimal cutting temperature (OCT) gel (Sakura, Torrance, CA, USA) at −30°C and cut into 20-μm sections, which were counterstained with 1 mg/mL DAPI for 5 min at 25°C. Then, the slides were observed using a fluorescence microscope (Leica, Germany).

### *In vivo* quantification of drug release

The glioma-bearing rats were divided into three groups and injected with free DOX, MSN-DOX-PDA, and MSN-DOX-PDA-NGR at a DOX dose of 5 mg/kg per animal. Then, 2 h post-treatment, the brain tissues were harvested after anesthetization and perfusion, and then divided into left (control) and right (tumor tissue containing) brain hemispheres. These brain samples were then homogenized and soaked in acidified ethanol (50% ethanol in 0.3N hydrochloric acid, HCl) for 24 h at 4°C to extract the DOX completely [42]. After drug extraction, the samples were centrifuged at 14000 rpm for 10 min using a freezing centrifuge (Sigma 3-30K, Heraeus Co., Germany). The concentration of DOX in the clear supernatant was analyzed using an HPLC method and expressed per gram of tissue. The DOX deposition in the liver tissues was also quantified to compare the temporary toxicity among those groups, using the same procedures above.

### *In vivo* antitumor and antiangiogenesis effects of dual-targeted NPs

The glioma-bearing rats were randomly divided into four groups of 16 each and injected with 100 μL of free DOX, DOX-MSN-PDA, MSN-DOX-PDA-NGR (DOX dose, 5 mg/kg), or saline via the tail vein on day 5, 8, 11, and 14 after the C6 cells implantation. On day 5 and 12 post-treatment, three rats from each group were euthanized. The brains were harvested, fixed with 10% neutral formalin at 4°C for 48 h, embedded in paraffin, and cut into 5-μm sections, which were stained with hematoxylin and eosin (H&E) using routine protocols and Tdt-mediated d-uridine triphosphate (UTP) nick-end labeling (TUNEL) using a kit, following the recommended protocol, to identify the histopathological changes. Simultaneously, CD31-specific staining was applied to assess the microvessel density (MVD) of the tumor tissues immunohistochemically. Ten tumor-bearing rats from each group were survival-monitored, and the survival data were analyzed using the log-rank test.

### *In vivo* biological safety study

Healthy adult male Sprague-Dawley rats were treated with MSN-PDA-NGR by tail vein injection (100 μL, 100 mg/kg) one dose every 3 days for 4 weeks. Rats treated with saline were used as controls. The body mass was recorded at day 1, 7, 14, 21, and 28 post-treatment. The main organs including the brain, heart, lungs, liver, spleen, and kidneys were harvested after the final treatment followed by H&E staining to examine the toxicity *in vivo*.

### Statistical analysis

All the experiments were performed in triplicate. The acquired data are presented as mean ± standard deviation (SD). The statistical significance was determined using ANOVA and *t*-test. *p* < 0.05 was considered significant.

## CONCLUSION

In this study, the MSN-PDA-NGR NPs were successfully established for the effective dual-targeting of BCEC-C6 and C6 cells. The drug carrier enhanced the cellular uptake of the NPs and the subsequent cytotoxicity significantly, as well as the BBB transportation ability *in vitro*. Furthermore, the dual-targeting drug delivery system exhibited a superior antiangiogenesis and antiglioma efficacy and negligible toxicity *in vivo*. In summary, these promising results indicate that the MSN-DOX-PDA-NGR could have great potential for the treatment of glioma.

## SUPPLEMENTARY MATERIALS AND METHODS, FIGURES


