# Supplementary material for: Asn-Gly-Arg-modified polydopamine-coated nanoparticles for dual-targeting therapy of brain glioma in rats
Source: Oncotarget. 2016 Sep 15;7(45):73681–96. doi: 10.18632/oncotarget.12047 (PMC5342007; doi:10.18632/oncotarget.12047)
Supplement: Supplementary file 1 [file oncotarget-07-73681-s001.pdf]

Asn-Gly-Arg-modified polydopamine-coated nanoparticles for dual-targeting therapy of brain glioma in rats

SUPPLEMENTARY DATA

MATERIALS AND METHODS

Primary cultures of BCEC and AC

Primary brain capillary endothelial cells (BCEC) were prepared from 4-week-old Sprague-Dawley male rats. The animal protocols were approved by the Animal Care and Use Committee of the Chongqing Medical University. In brief, the rat brains were harvested under ether anesthesia and stored in phosphate-buffered saline (PBS) on ice followed by separation of the cortices under aseptic conditions. Then, the cortices were chopped into approximately 1-mm^3 pieces gently, homogenized, and treated with a mixture of collagenase (1 mg/mL) and DNAse (30 μ/mL) for 1 h at 37°C. The microvessel enriched pellet was obtained by centrifugation (1500 rpm, 15 min, 4°C) with 20% bovine serum albumin (BSA) and subsequently incubated in collagenase/dispase (1 mg/mL) mixed with DNAse (20 u/mL) for 1 h at 37°C. Then, the microvessel fragments were separated by centrifugation at 4°C (800 rpm, 30 min) in a non-continuous Percoll gradient (70%, 60%, 50%, 40%, 30%, and 20%). The capillary clusters between 30% and 50% density gradient were obtained and washed with PBS, seeded in six-well plates coated with type IV collagen (5 μg/cm^2), and maintained in complete culture medium containing puromycin (4 μg/mL) for 2 days to kill the contaminating cell types without inhibiting the growth of the BCEC cells [1].

Primary astrocytes (AC) were prepared from the cortices of 3-day-old Sprague-Dawley male rats. Briefly, the homogenized cortices mentioned above were incubated with trypsin (0.25%) at 37°C for 30 min and filtered through a 10-μm nylon filter, and the astrocytes were obtained and cultured in Dulbecco's modified Eagle's medium (DMEM, high glucose) supplemented with 10% fetal bovine serum (FBS and 1% astrocyte growth supplement.

Transendothelial electrical resistance (TEER)

The TEER (Ω×cm^2) was measured using a TEER instrument (Millicell-RES, Millipore, USA). Final resistance values were calculated by subtracting the resistance values of the empty filter and multiplying by the surface area of the insert (4.5 cm^2).

SUPPLEMENTARY REFERENCE

1. Perrière N, Ph. D, Garcia E, Debray M, - P. AJ, Couvreur P, - M. SJ, Temsamani J, - O. CP. Puromycin-based purification of rat brain capillary endothelial cell cultures. Effect on the expression of blood-brain barrier-specific properties. Journal of Neurochemistry. 2005; 93:279-289.

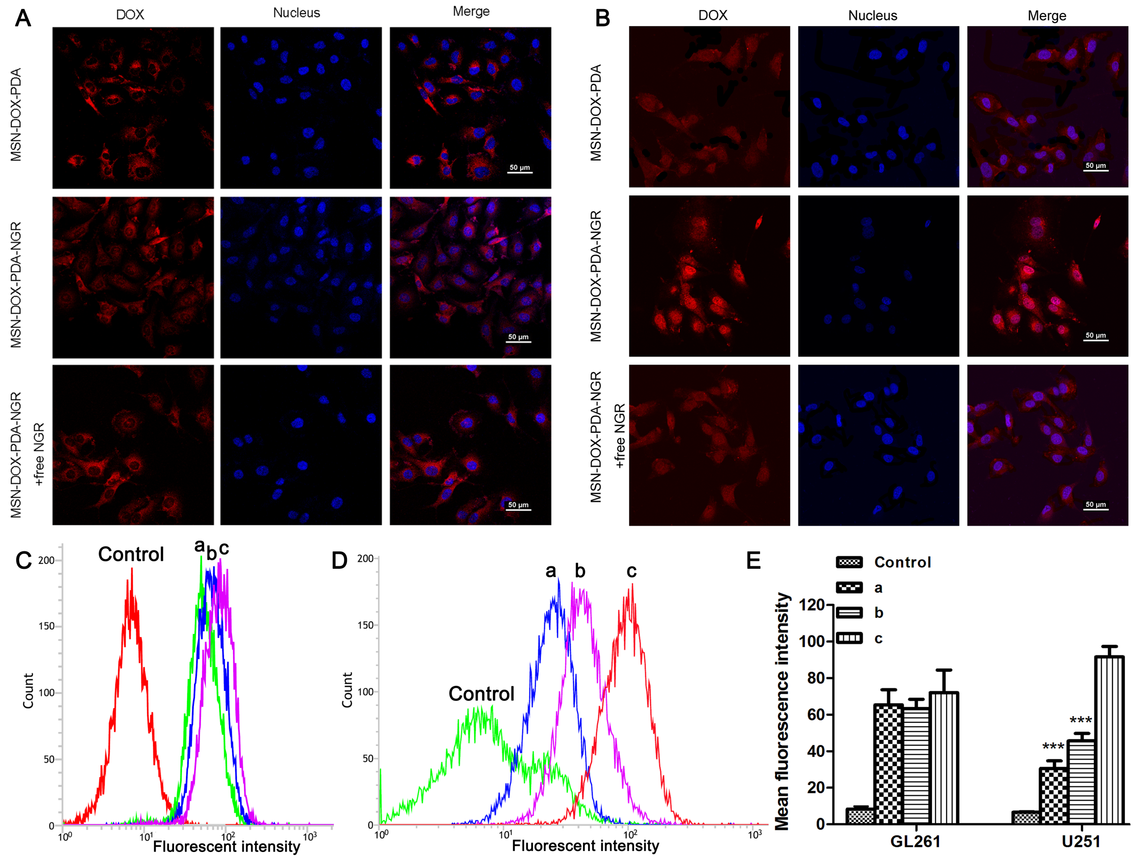

Supplementary Figure S1: Qualitative and quantitative analysis of drug-loaded NPs in GL261 and U251 cells. Confocal images of **A.** GL261 and **B.** U251 cells after treatment with different DOX formulations for 4 h. Flow cytometric analysis of **C.** GL261 and **D.** U251 cells after treatment with different DOX formulations for 2 h. **E.** Quantitative analysis of mean fluorescence intensity in different DOX formulation group. Cell nuclei were stained using DAPI (blue) and DOX (red). (a) MSN-DOX-PDA, (b) MSN-DOX-PDA-NGR + free NGR, and (c) MSN-DOX-PDA-NGR. $^{***}p < 0.001$ compared with c (n = 3).

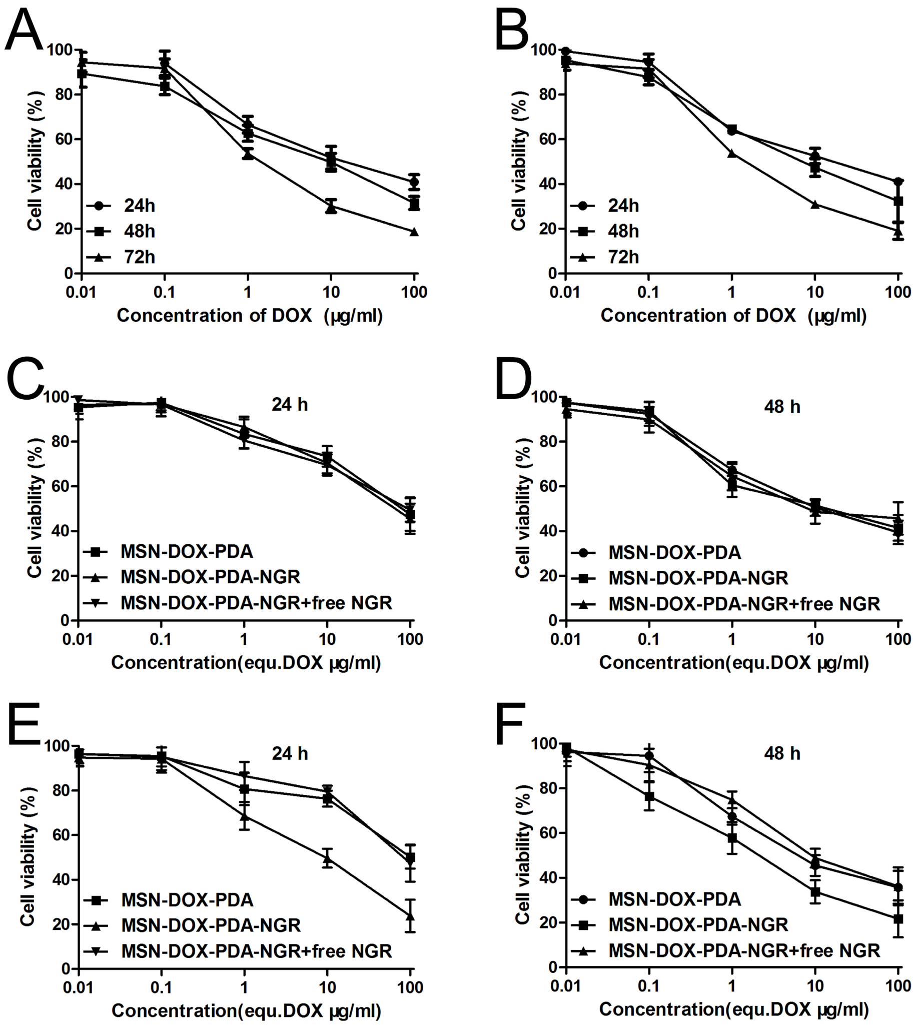

Supplementary Figure S2: Cell viability following treatment with various NPs. Viability of **A.** GL261 and **B.** U251 cells after treatment with free DOX. Viability of GL261 cells after treatment with different DOX formulations for **C.** 24 and **D.** 48 h. Viability of U251 cells after treatment with different DOX formulations for **E.** 24 and **F.** 48 h.
